# In Silico Prediction of Bioactive Peptides From *Lactococcus lactis* SwR14: Evaluation of a Potential Probiotic Candidate

**DOI:** 10.1155/ijm/2089550

**Published:** 2026-10-21

**Authors:** Yuliana Tandi Rubak, Herianus J. D. Lalel, Maxs Urias Ebenhaizar Sanam, Fenny Amilia Mahara, Dandy Yusuf

**Affiliations:** ^1^ Department of Agrotechnology Universitas Nusa Cendana Kupang East Nusa Tenggara Indonesia undana.ac.id; ^2^ Department of Animal Diseases Sciences and Veterinary Public Health Universitas Nusa Cendana Kupang East Nusa Tenggara Indonesia undana.ac.id; ^3^ Research Center for Food Technology and Processing National Research and Innovation Agency (BRIN) Gunungkidul Special Region of Yogyakarta Indonesia brin.go.id; ^4^ Research Center for Applied Microbiology National Research and Innovation Agency (BRIN) Cibinong West Java Indonesia brin.go.id; ^5^ Research Collaboration Center for Traditional Fermentation Surakarta Central Java Indonesia

**Keywords:** alpha-glucosidase, antioxidant, bioactive peptide, cholesterol, folate, *Lactococcus lactis*

## Abstract

Lactic acid bacteria (LABs) are widely recognized for their nutritional and therapeutic potential, primarily due to their ability to produce bioactive peptides during fermentation. In this study, *Lactococcus lactis* strains, potential probiotics from Sui Wu′u, a traditional fermented meat from Bajawa, East Nusa Tenggara, Indonesia, were evaluated for folate biosynthesis, cholesterol‐lowering activity, *α*‐glucosidase inhibition, and antioxidant capacity, and their association with bioactive peptide production in milk was investigated. Among the isolates, SwR14 and SwR16 were able to grow under folate‐depleted conditions, indicating folate autotrophy, with SwR14 producing the highest extracellular folate concentration in folate‐free medium (12.40 ng/mL). All isolates exhibited moderate in vitro cholesterol reduction (14.28%–20.94%) and *α*‐glucosidase inhibitory activity (25.94%–33.96%), with no significant interstrain differences. Antioxidant activity assessed using DPPH, ABTS, and FRAP assays showed assay‐dependent variability, with SwR14 demonstrating the most efficient DPPH radical scavenging activity based on the lowest IC_50_ value. Peptides from milk fermented by *L. lactis* SwR14 were then identified via liquid chromatography–high‐resolution tandem mass spectrometry (LC‐HRMS/MS) and profiled using the BIOPEP‐UWM database, revealing 49 peptide sequences with 47 distinct biological activities. ACE and DPP‐IV inhibitory activities were detected in all sequences, whereas *α*‐glucosidase inhibitory and antioxidant activities were predicted in 33 and 31 sequences, respectively, consistent with the in vitro results. Several peptide sequences emerged as promising candidates for antioxidant and *α*‐glucosidase inhibitory activity on the basis of their molecular weight and hydrophobic amino acid composition, suggesting that milk fermentation by *L. lactis* SwR14 promotes bioactive peptide generation despite its weak proteolytic phenotype. These findings highlight *L. lactis* SwR14 as a promising functional strain for the development of bioactive peptide–enriched dairy products. Future studies should explore peptide bioactivity mechanisms and validate their efficacy through in vivo studies.

## 1. Introduction

Lactic acid bacteria (LABs) are key fermentation microorganisms that increase both the nutritional and functional properties of fermented foods and beverages [1]. During fermentation, LABs produce a wide range of metabolites, including lactic acid, bioactive peptides, exopolysaccharides (EPSs), bacteriocins, enzymes, short‐chain fatty acids, and vitamins [1–3]. These metabolites contribute to various health benefits, such as antioxidant, anti‐inflammatory, hypoglycemic, immune‐boosting, antitumor, antiobesity, bone health‐promoting, antihypertensive, lactose intolerance‐reducing, and antidiabetic effects [1, 2].

Among LAB metabolites, bioactive peptides derived from fermented milk have drawn considerable attention for their potential health benefits [4–6]. These peptides, which are composed of short amino acid chains (2–20 residues), are defined by their unique composition, sequence, and structure [7]. They originate from inactive precursor proteins that are activated through proteolytic activity during LAB fermentation [8]. Thus, the proteolytic system of LAB, which involves proteinases and peptidases, plays a crucial role in breaking down proteins into peptides to meet their metabolic nutritional needs, with the concurrent release of bioactive peptides [9, 10].

LAB strains with high proteolytic activity are closely linked to the production of bioactive peptides, offering a cost‐effective source of peptides with specific bioactivities [9–11]. However, the proteolytic activity of LAB varies between strains and depends on enzyme‐substrate specificity, allowing them to produce a wide range of bioactive peptides [9, 11]. For example, research by Picon et al. [12] revealed that *Lactococcus lactis* strains exhibit differences in proteolysis and peptidolysis activities. Interestingly, some nonproteolytic strains, such as ESI292, ESI392, ESI413, and ESI441, presented significant peptidolytic activity and were capable of breaking down peptides into free amino acids. Despite lacking proteolytic activity, these strains can thrive in fermentation processes due to their peptide transport systems (e.g., Opp and Dpp), which enable growth on substrates deficient in essential amino acids. This variability in proteolytic and peptidolytic activities across *L. lactis* strains presents opportunities for the production of diverse bioactive peptides.

Bioactive peptides produced by *L. lactis* during milk fermentation exhibit diverse biological activities, including antioxidant, antidiabetic, and cholesterol‐lowering effects [13]. These activities have attracted considerable interest due to their potential roles in alleviating oxidative stress, regulating glucose metabolism, and lowering cholesterol levels. In particular, the antioxidant capacity of LAB has been associated with protection against oxidative stress–related disorders [14], whereas *α*‐glucosidase inhibitory activity has been linked to hypoglycemic and antidiabetic potential [15]. In addition, LAB and their metabolites have been reported to contribute to cholesterol reduction and cardiovascular health through several mechanisms, including cholesterol binding and modulation of lipid metabolism [16]. Besides these functional attributes, microbial folate production offers a natural approach to improve dietary folate intake. Recent evidence suggests that probiotic bacteria, including *L. lactis*, may contribute to enhanced folate status and help prevent folate deficiency‐related disorders, such as anemia and hyperhomocysteinemia [17]. Therefore, *L. lactis* strains capable of simultaneously producing folate and exhibiting antioxidant, *α*‐glucosidase inhibitory, and cholesterol‐binding activities may represent promising candidates for the development of multifunctional fermented foods.

Although members of the genus *Lactococcus* were historically not regarded as probiotic bacteria due to their presumed absence as natural intestinal inhabitants [18], increasing evidence indicates that certain strains exhibit strain‐specific probiotic potential. For instance, Divya and Nampoothiri [19] demonstrated that *L. lactis* CM28 isolated from cow′s milk possessed probiotic characteristics while simultaneously acting as an efficient folate producer, enabling successful skim milk fortification with high folate stability during cold storage. In line with these findings, Kondrotienė et al. [20] reported that *L. lactis* strains naturally present in raw and fermented milk exhibited notable in vitro probiotic traits, including tolerance to acidic and bile conditions, cholesterol‐related properties, and favorable technological performance as starter cultures.

Beyond dairy‐origin strains, Rubak et al. [21] reported that four *L. lactis* isolates (SwR14, SwR16, SwR23, and SwR24) obtained from Sui Wu′u, a traditional fermented pork product from East Nusa Tenggara, Indonesia, exhibited key in vitro probiotic characteristics, including acid and bile tolerance, antagonistic activity against pathogenic bacteria, and acceptable antibiotic susceptibility profiles. In addition, amino acid profiling of Sui Wu′u revealed a rich composition of essential and nonessential amino acids, indicating active microbial involvement in protein metabolism during fermentation [22]. Since proteolytic activity is closely associated with the release of bioactive peptides [9–11], these meat‐origin isolates represent promising candidates for evaluating their ability to utilize milk proteins as precursors for peptide generation.

Although *L. lactis* strains isolated from dairy products have been extensively investigated, information regarding the multifunctional properties and peptide‐producing potential of indigenous meat‐origin strains remains limited. Therefore, exploring these isolates in dairy fermentation systems may expand the diversity of functional starter candidates and provide insight into strain‐specific traits associated with bioactive peptide production. This study is aimed at evaluating the nutritional and functional properties in the four *L. lactis* isolates (SwR14, SwR16, SwR23, and SwR24) from Sui Wu′u, with the hypothesis that these meat‐origin strains possess strain‐specific technofunctional traits that could be exploited for the development of functional dairy products. This work provides insight into the strain‐specific functional attributes of meat‐origin *L. lactis* and highlights their potential application in developing novel functional dairy products, where diverse bioactivities and peptide profiles are desirable.

## 2. Materials and Methods

### 2.1. Microbial Strains and Cultivation Conditions

This study used *L. lactis* strains (SwR14, SwR16, SwR23, and SwR24) previously isolated from Sui Wu′u, a traditional fermented meat from Bajawa in East Nusa Tenggara, Indonesia, known for its probiotic potential [21]. Before application, each isolate was subcultured twice in commercial de Man–Rogosa–Sharpe broth (MRSB; CM0359, Oxoid Ltd., Basingstoke, United Kingdom), prepared according to the manufacturer′s instructions. Both subcultures were incubated statically under aerobic conditions at 37°C for 24 h, and the resulting cultures were subsequently used as inocula for all experiments to ensure consistent cultivation conditions.

### 2.2. Preparation of Cell‐Free Supernatant (CFS) and Standard LAB Suspension

Unless otherwise specified, all preparation of CFS and bacterial suspensions was performed as described in this section. LAB isolates were cultivated in MRSB at 37°C for 24 h. The cultures were centrifuged at 8000 × g for 10 min at 4°C (Mikro 22R, Hettich, Tuttlingen, Germany). For assays requiring viable bacterial cells (cholesterol‐lowering and folate production assays), harvested pellets were washed twice with phosphate‐buffered saline (PBS; pH 6.8; BR0014G, Oxoid Ltd., Basingstoke, United Kingdom) and resuspended in the same solution.

For assays requiring CFS, the culture supernatant was filtered through a 0.22 *μ*m polyethersulfone (PES) membrane syringe filter (Minisart, Sartorius, Germany). The resulting CFS was used for antioxidant assays, whereas a pH‐adjusted aliquot was used for the *α*‐glucosidase inhibitory assay. For the *α*‐glucosidase inhibitory assay, the pH of the CFS was adjusted to 7.4 using 1 N NaOH to ensure optimal enzyme reaction conditions. For antioxidant assays, the CFS was used without pH adjustment.

### 2.3. Assessment of Nutritional and Functional Properties

#### 2.3.1. Extracellular Folate Production

The extracellular folate production of the LAB isolates was evaluated following the method described by Mahara et al. [23], employing a microbiological assay based on the growth response of folate‐dependent indicator bacteria in folate‐free culture media. A standardized LAB cell suspension was prepared as described in Section 2.2, and the optical density at 600 nm (OD_600_) was measured using a spectrophotometer (Genesys 10S, Thermo Fisher Scientific, Waltham, Massachusetts, United States). Prior to the experiments, OD_600_ measurements were calibrated against viable counts determined by serial dilution and plating on MRS agar followed by incubation at 37°C for 48 h. Based on this calibration, the cell suspension was diluted with PBS to obtain a standardized inoculum with an estimated cell density of approximately 5–6 log CFU/mL.

A 2% inoculum (v/v) was cultivated in folic acid casei medium (FACM; M543, HiMedia Laboratories, Mumbai, India), followed by incubation for 24 h at 37°C. FACM is formulated to be free of folic acid and glutamic acid, while containing *p*‐aminobenzoic acid (*p*‐ABA) to support de novo folate biosynthesis under folate‐depleted conditions [24]. This process was conducted twice to ensure optimal growth.

In addition, the inoculum was cultured in MRSB as a control medium, which is considered folate‐rich due to the presence of yeast extract, a natural source of folate and its biosynthetic precursors [24], under the same conditions. The growth absorbances of the cultures were measured with a UV–visible spectrophotometer (Cary 60 UV‐Vis, Agilent Technologies, Santa Clara, California, United States) set to 580 nm. The isolates exhibiting the highest OD_580_ values were selected for further assessment of extracellular folate extraction and quantification.

Extracellular folate was extracted by centrifugation of the culture, followed by filtration through a 0.2 *μ*m nylon membrane filter (ANPEL Laboratory Technologies Inc., Shanghai, China). Folate quantification was performed using the VitaFast Folic Acid Test Kit (P1001, R‐Biopharm, Pfungstadt, Germany). This kit is a microbiological assay based on the growth of folate‐dependent indicator bacteria, with a detection range of 1.6–12.8 ng/mL, a sensitivity of 0.1 ng/mL, and an incubation period of 48 h at 37°C. Folate standards provided in the kit were used to construct a standard curve. Filtered culture supernatants and standards were added to wells of a microtiter plate coated with the indicator bacteria, incubated in the dark for 48 h, and growth was measured using a microtiter plate reader (Multiskan GO, Thermo Scientific, Waltham, Massachusetts, United States) at 595 nm. Folate concentrations were calculated by reference to the standard curve.

#### 2.3.2. Cholesterol‐Lowering Effects

Cholesterol‐lowering activity was evaluated using viable bacterial cells prepared as described in Section 2.2 under three physiological states, including the exponential (growing), stationary (resting), and death phases, according to the method described by Yusuf et al. [25]. These physiological states were included to evaluate the contribution of different physiological states of LAB to cholesterol removal and to distinguish metabolically mediated cholesterol removal from passive interactions between cholesterol and the bacterial cell surface. LAB cultures were initially grown in MRSB supplemented with 0.5% (w/v) bile salt (HiMedia Laboratories, Mumbai, India) and incubated at 37°C for 24 h.

For the growing cell assay, cultures were centrifuged, and the resulting pellets were recultured in MRSB containing 0.5% bile salt and 100 ppm cholesterol (PEG‐600; Sigma‐Aldrich, St. Louis, Missouri, United States) at 37°C for 24 h. For the resting cell assay, harvested cell pellets were resuspended in PBS (pH 6.8) supplemented with 0.5% bile salt and 100 ppm cholesterol and incubated under the same conditions. For the dead cell assay, cell suspensions in PBS were heat‐treated at 121°C for 15 min, followed by centrifugation. The resulting pellets were then resuspended in MRSB supplemented with 0.5% bile salt and 100 ppm cholesterol and incubated at 37°C for 24 h.

After incubation, samples from all treatments were centrifuged, and the supernatants were collected for cholesterol quantification using a colorimetric *o*‐phthalaldehyde (OPA) method (79760, Sigma‐Aldrich, St. Louis, Missouri, United States). Absorbance was measured at 550 nm using a UV–visible spectrophotometer (Cary 60 UV‐Vis, Agilent Technologies, Santa Clara, California, United States). Cholesterol concentration was calculated from a standard curve, and cholesterol reduction (%) was determined using the following equation:

Cholesterol reduction %=A01−AA0×100,

where *A*_0_ represents the initial cholesterol concentration in the medium (ppm) and *A*_1_ represents the residual cholesterol concentration after incubation (ppm).

#### 2.3.3. *α*‐Glucosidase Inhibitory Activity

The *α*‐glucosidase inhibitory activity was determined according to the method described by Son et al. [26], with minor modifications. For the assay, 25 *μ*L of CFS (prepared as described in Section 2.2) was mixed with 150 *μ*L of phosphate buffer (pH 7.4, Oxoid Ltd., Basingstoke, United Kingdom) and 75 *μ*L of 20 mM *p*‐nitrophenyl *α*‐D‐glucopyranoside (Sigma‐Aldrich, St. Louis, Missouri, United States) as the substrate. The mixture was preincubated at 37°C for 10 min. Subsequently, 50 *μ*L of *α*‐glucosidase solution (0.2 U/mL, from *Saccharomyces cerevisiae*, Sigma‐Aldrich, St. Louis, Missouri, United States) was added, and the reaction mixture was further incubated at 37°C for 10 min. The reaction was terminated by the addition of 1 mL of 0.1 M Na_2_CO_3_ (Merck, Darmstadt, Germany).

The absorbance was measured at 405 nm using a microplate reader (iMark, Bio‐Rad Laboratories, Hercules, California, United States). The percentage of *α*‐glucosidase inhibitory activity was calculated using the following equation:

α−Glucosidase inhibitor activity %=1−C−DA−B ×100,

where *A* represents the absorbance of the control reaction containing *α*‐glucosidase without sample, *B* represents the absorbance of the blank (sterile deionized water), *C* represents the absorbance of the reaction containing both *α*‐glucosidase and sample, and *D* represents the absorbance of the sample without enzyme (used to correct background absorbance).

#### 2.3.4. Antioxidant Activity

##### 2.3.4.1. DPPH Radical Scavenging Activity

DPPH radical scavenging activity was determined according to Fitrianto et al. [27] with minor modifications. CFS was serially diluted in methanol (3.125%–100%, v/v). A total of 100 *μ*L of sample was mixed with 100 *μ*L of DPPH solution (0.04 mg/mL) in a 96‐well plate and incubated for 20 min in the dark at room temperature. Absorbance was measured at 517 nm, with ascorbic acid used as a positive control. Radical scavenging activity was calculated as percentage inhibition:

Inhibition %=Acontrol−AsampleAcontrol×100.

##### 2.3.4.2. ABTS Radical Scavenging Activity

ABTS radical scavenging activity was performed according to Fitrianto et al. [27] with slight modifications. The ABTS^+^ radical was generated by reacting ABTS (7 mM) with potassium persulfate (2.45 mM) and incubating for 16 h in the dark, followed by dilution to an absorbance of 0.70 ± 0.02 at 734 nm. Then, 40 *μ*L of diluted CFS (3.125%–100%, v/v) was mixed with 160 *μ*L ABTS^+^ solution and incubated for 10 min in the dark. Absorbance was recorded at 734 nm. Radical scavenging activity was expressed as percentage inhibition using the same equation as above.

##### 2.3.4.3. Ferric Reducing Antioxidant Power (FRAP) Assay

FRAP assay was conducted according to Fitrianto et al. [27] and Wahyono et al. [28] with modifications. FRAP reagent was freshly prepared by mixing TPTZ, FeCl_3_, and acetate buffer (1:1:10, v/v/v). Then, 20 *μ*L of CFS was mixed with 200 *μ*L FRAP reagent and incubated for 45 min in the dark at room temperature. Absorbance was measured at 593 nm. Results were expressed as FeSO_4_ equivalents based on a standard calibration curve.

### 2.4. Proteolytic Activity

#### 2.4.1. Qualitative Proteolytic Activity on Skim Milk Agar

Proteolytic activity of *L. lactis* isolates was evaluated using qualitative and quantitative approaches. Preliminary screening was conducted on skim milk agar to assess caseinolytic activity. Briefly, the isolates were grown in MRSB at 37°C for 24 h, streaked onto skim milk agar plates (HiMedia Laboratories, Mumbai, India), and incubated at 37°C for 48 h. The formation of clear zones surrounding bacterial colonies was considered indicative of proteolytic activity, and the diameters of the clearance zones were measured as previously described [29, 30].

#### 2.4.2. Quantitative Proteolytic Activity During Milk Fermentation

Quantitative proteolytic activity during milk fermentation was determined by measuring the release of free amino groups using the OPA assay, following the method of Panchal et al. [31] with modifications. An aliquot of 1 mL of the LAB suspension (prepared as described in Section 2.2) was inoculated into 10 mL of sterile skim milk (7%, w/v) and incubated at 37°C for 48 h. Following fermentation, the samples were centrifuged to obtain the supernatant. Protein precipitation was performed using trichloroacetic acid (TCA) at a final concentration of 12% (w/v), achieved by mixing the supernatant with an equal volume of 24% (w/v) TCA. This modification was applied to ensure complete precipitation of milk proteins and to obtain a clear filtrate suitable for OPA analysis. The mixtures were incubated at room temperature for 10 min and subsequently centrifuged.

A volume of 200 *μ*L of the clear supernatant was reacted with 3 mL of OPA reagent, vortexed, and incubated at room temperature for 2 min. Absorbance was measured at 340 nm using a microplate reader (iMark, Bio‐Rad Laboratories, Hercules, California, United States). Nonfermented skim milk subjected to the same experimental conditions was used as a negative control, whereas the OPA reagent without sample served as the blank.

#### 2.4.3. Detection of *prtP* Gene

The presence of the *prtP* gene encoding cell‐envelope proteinase (CEP) was examined to support the proteolytic characterization of *L. lactis* isolates at the molecular level. Genomic DNA was extracted from overnight LAB cultures using the Presto Mini gDNA Bacteria Kit (Geneaid Biotech Ltd., Taiwan) according to the manufacturer′s instructions. The concentration and purity of the extracted DNA were determined. Polymerase chain reaction (PCR) amplification of *prtP* genes was performed based on the method described by Yonezawa et al. [32], with minor modifications. Two sets of primers were used to specifically detect *prtP* alleles encoding Group b and Group h proteinases. For Group b, primers GBf (GCAAATACGGTGACGGCTGCGA) and GB2r (TGAGCTTATAATAGGTCTTCTTCC) were used, whereas primers GHf (CAAATACGGTGACGGCTGCTAA) and GH2r (TAGCATTATAATAGGTCTTCGTCA) were employed for Group h detection. PCR reactions were carried out using a Mastercycler X50s thermal cycler (Eppendorf, Hamburg, Germany). Amplification conditions consisted of an initial denaturation at 94°C, followed by 25 cycles of denaturation at 94°C for 10 s, primer annealing at gradient temperatures of 46°C–50°C for *prtP* Group b and 45°C–49°C for *prtP* Group h for 30 s, and extension at 72°C for 1 min. A final extension step was performed at 72°C for 5 min, after which the reactions were cooled to 4°C.

The PCR products were analyzed by electrophoresis on a 1.0% (w/v) agarose gel (Simply Agarose, GeneDireX, Taiwan) stained with a nucleic acid staining dye (GelRed Nucleic Acid Stain, Merck Millipore, United States) and visualized using a gel documentation system (Bio‐Rad Laboratories, Hercules, California, United States). The expected amplicon size for prtP was approximately 2.7 kb.

### 2.5. Peptide Production in Fermented Milk

The isolate selected for subsequent peptide production was chosen based on an overall evaluation of its functional characteristics, including folate autotrophy, extracellular folate production, cholesterol‐lowering activity, *α*‐glucosidase inhibitory activity, and antioxidant capacity, rather than on superiority in a single parameter. The selected LAB culture was initially cultivated in MRSB at 37°C for 24 h, and the resulting 24‐h culture was used as the starter inoculum. Skim milk (11%, w/v, NZWP Ltd., New Zealand) was pasteurized at 95°C for 10 min, inoculated with 2% (v/v) of the LAB culture, and incubated at 37°C for 24 h. This adaptation step was repeated twice to ensure stable growth and optimal performance of the isolate as a starter culture.

Subsequently, 2% (v/v) of the adapted starter culture was reinoculated into freshly prepared skim milk medium and incubated at 37°C for 24 h. After fermentation, samples were analyzed for LAB viable counts, titratable acidity, and soluble protein content. LAB counts were determined on MRSA, titratable acidity was measured by acid‐base titration, and soluble protein content was quantified using the Bradford method.

For peptide analysis, fermented milk samples were immediately centrifuged. The resulting supernatant was collected and used for partial purification and subsequent peptide identification.

### 2.6. Identification of Peptides by Mass Spectrometry (MS)

The supernatant was subjected to sequential ultrafiltration using Amicon Ultra‐4 mL centrifugal filters (Merck, Darmstadt, Germany) with molecular weight cutoffs (MWCO) of 10 and 3 kDa, as previously described [33]. Three fractions corresponding to nominal molecular weight ranges of > 10, 3–10, and < 3 kDa were collected for peptide characterization. Peptide characterization was performed at the LPPT Laboratory, Universitas Gadjah Mada, following the method of Ningsih et al. [34] via LC‐HRMS. The system consisted of a Thermo Scientific Dionex Ultimate 3000 RSLCnano UHPLC coupled with a Thermo Scientific Q Exactive high‐resolution mass spectrometer.

Sample fractions (5 *μ*L) were injected into the LC‐HRMS system and initially trapped on a PepMap RSLC‐C18 column (75 *μ*m × 150 mm, 3 *μ*m particle size, 100 Å pore size; Thermo Scientific). Samples were washed for 6 min using 98% Solvent A (water with 2% acetonitrile, 0.1% formic acid) and 2% Solvent B (80% acetonitrile in water, 0.1% formic acid) at a flow rate of 5 *μ*L/min. Peptide separation was subsequently performed using a linear gradient from 10% to 95% Solvent B over 90 min at a flow rate of 0.3 *μ*L/min.

MS detection was carried out in full‐MS/dd‐MS^2^ positive ion scanning mode, with full‐MS resolution set at 70,000 and dd‐MS^2^ resolution at 17,500. The mass–charge (m/z) range analyzed was 150–2000. Peptide data were processed using Proteome Discoverer software Version 2.2 (Thermo Fisher Scientific, Waltham, Massachusetts, United States). Peptide identification was performed against the UniProt/Swiss‐Prot protein database using no‐enzyme specificity, with precursor and fragment mass tolerances set to 10 ppm and 0.02 Da, respectively. Oxidation of methionine was considered a variable modification, and peptide‐spectrum matches were filtered at a false discovery rate (FDR) threshold of 1%.

### 2.7. Prediction of Peptide Function via BIOPEP‐UWM

The functional properties of the identified peptide sequences were predicted using the BIOPEP‐UWM database, following the method described by Cui et al. [35]. The analysis focused on key biological activities, including cholesterol‐lowering, *α*‐glucosidase inhibition, and antioxidant potential. Peptide sequences were uploaded to the BIOPEP‐UWM database under the “bioactive peptides” module, and the “analysis” and “for your sequence” functions were used to evaluate the potential biological activity profiles. Functional activity profiles were generated automatically, and peptides containing repeated fragments with a ratio > 30% were classified as potential bioactive peptides.

### 2.8. Statistical Analysis

All experiments were performed in triplicate (*n* = 3). Data were analyzed using SPSS Version 20.0 (IBM Corp., Armonk, New York, United States) and are presented as mean ± standard deviation (SD). Prior to statistical analysis, data normality was assessed using the Shapiro–Wilk test, whereas homogeneity of variance was evaluated using Levene′s test.

Differences in OD_580_ values among strains within each growth medium (FACM and MRSB) were analyzed using one‐way ANOVA followed by Tukey′s post hoc test. Comparisons of OD_580_ values between FACM and MRSB for each strain were performed using paired *t*‐tests. Differences in extracellular folate production were analyzed using one‐way ANOVA followed by Tukey′s post hoc test. Statistical significance was defined as *p* < 0.05.

## 3. Results and Discussion

### 3.1. Nutritional and Functional Properties of *L. lactis* Strains

#### 3.1.1. Extracellular Folate Production

Among the four *L. lactis* isolates examined, SwR14 and SwR16 exhibited measurable growth in folate‐free medium (FACM), whereas SwR23 and SwR24 showed only negligible growth (Figure 1A). Notably, SwR14 exhibited significantly greater growth than the other three isolates in FACM (*p* < 0.05), whereas SwR16 showed intermediate growth but remained capable of sustaining growth under folate‐depleted conditions. The absorbance levels of SwR14 and SwR16 in FACM were lower than the OD_580_ values (> 1.0) reported for folate‐producing LAB isolates grown in folate‐free media [23]. Such variability likely reflects strain‐specific differences. Nevertheless, the ability of SwR14 and SwR16 to grow in FACM suggests an intrinsic folate biosynthetic capacity, supporting their classification as folate‐autotrophic LAB. In contrast, SwR23 and SwR24 exhibited growth exclusively in folate‐containing MRSB, indicating a dependence on exogenous folate and supporting their classification as folate‐consuming bacteria.

**Figure 1:**
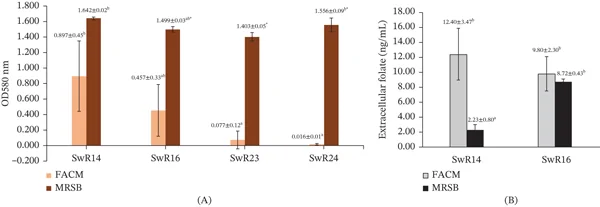
Growth performance and extracellular folate production of *Lactococcus lactis* isolates. Data are presented as mean ± SD (*n* = 3). (A) Growth of *L. lactis* isolates in folic acid casei medium (FACM) and de Man, Rogosa, and Sharpe broth (MRSB) after 24 h of incubation, expressed as OD_580_. Different lowercase letters indicate significant differences among strains within the same medium (one‐way ANOVA followed by Tukey′s post hoc test, *p* < 0.05). Asterisks (∗) indicate significant differences between FACM and MRSB within the same strain (paired *t*‐test, *p* < 0.05). (B) Extracellular folate concentrations produced by *L. lactis* SwR14 and SwR16 in FACM and MRSB after 24 h of incubation. Folate values were corrected for the baseline folate content of MRSB. Different lowercase letters indicate significant differences among treatment groups (one‐way ANOVA followed by Tukey′s post hoc test, *p* < 0.05).

Based on their growth performance in FACM, SwR14 and SwR16 were further evaluated for extracellular folate production in both folate‐free (FACM) and folate‐containing (MRSB) media. These assessments are aimed at examining not only their ability to synthesize folate de novo but also their potential to overproduce folate in nutritionally rich environments, which is relevant for application in diverse food matrices [23, 24].

After 24 h of incubation, SwR16 produced comparable extracellular folate concentrations in FACM (9.80 ng/mL) and MRSB (8.72 ng/mL) (Figure 1B), indicating that its folate production capacity was not markedly influenced by the initial presence of folate in the growth medium. This behavior suggests that SwR16 may function as a folate overproducer, maintaining consistent folate synthesis regardless of external folate availability, which is a desirable trait for functional food applications. In contrast, SwR14 produced significantly higher extracellular folate levels in FACM (12.40 ng/mL) than in MRSB (2.23 ng/mL) (Figure 1B), suggesting that folate limitation may stimulate folate biosynthesis in this strain. Particularly, the folate concentration produced by SwR14 in FACM exceeded levels reported for other *L. lactis* strains under comparable conditions [36–38]. Consistent with previous reports, folate production among LAB is highly strain‐dependent and influenced by cultivation parameters, including incubation time and medium composition [19, 24]. Therefore, further optimization of fermentation conditions is warranted to maximize folate production by these isolates.

#### 3.1.2. Cholesterol‐Lowering Activity

The in vitro assay demonstrated cholesterol reduction by all four *L. lactis* isolates in the growth media, with percentages ranging from 14.28% to 20.94% for growing cells, 5.00% to 12.29% for resting cells, and 10.25% to 18.75% for dead cells (Table 1). Variability was observed, particularly for dead cells, where the calculated percentage reduction is highly sensitive to small changes in absorbance values. As a result, minor differences in optical density may lead to relatively large variations in calculated cholesterol reduction percentages. In contrast, cholesterol reduction by growing and resting cells showed more consistent trends, as reflected by lower SD values.

**Table 1:** Cholesterol reduction by growing, resting, and dead *L. lactis* isolates from Sui Wu′u.

Isolates	Cholesterol reduction (%)		
	Growing cells	Resting cells	Dead cells
*L. lactis* SwR14	20.94 ± 13.17^a^	10.63 ± 0.41^a^	17.50 ± 15.91^a^
*L. lactis* SwR16	14.35 ± 3.54^a^	5.38 ± 0.77^a^	10.25 ± 3.18^a^
*L. lactis* SwR23	14.28 ± 3.71^a^	12.29 ± 6.31^a^	12.75 ± 30.41^a^
*L. lactis* SwR24	18.10 ± 2.65^a^	9.28 ± 3.22^a^	18.75 ± 14.14^a^

*Note:* Values are presented as mean and standard deviation. Different lowercase superscripts in the same column indicate significant differences (*p* < 0.05).

Despite the observed variability, statistical analysis revealed no significant differences among the isolates within each cell condition. The data suggest that cholesterol reduction in this study was more consistently observed in metabolically active cells, supporting the relevance of growing and resting cells as the main contributors to cholesterol‐lowering activity.

The cholesterol‐lowering potential of *L. lactis* has also been demonstrated both in vitro [39, 40] and in vivo [41]. However, the cholesterol‐reducing activity observed in this study was lower than that reported for other *L. lactis* strains, as well as strains from *Lactobacillus* and *Enterococcus* [42, 43], indicating that cholesterol‐reducing efficacy varies considerably even among *L. lactis* strains. Overall, cholesterol reduction by LAB is strain‐dependent and influenced by the physiological state of the cells [25, 44]. Evaluating growing, resting, and dead cells provides insight into the possible mechanisms underlying cholesterol removal. Growing cells are generally considered to remove cholesterol predominantly through metabolically mediated assimilation or incorporation into the cell membrane during active growth, whereas cholesterol reduction by dead cells reflects nonmetabolic mechanisms, primarily passive adsorption to the bacterial cell surface or cell wall [44, 45]. Resting cells represent an intermediate physiological state between actively growing and nonviable cells, although the mechanisms responsible for cholesterol removal under this condition remain less well understood. In the present study, the consistently higher cholesterol reduction observed in growing cells than in resting and dead cells suggests that active metabolism plays a major role in cholesterol removal by the tested isolates, whereas passive adsorption likely contributes to a lesser extent. This interpretation is consistent with previous observations reported for LAB, including *L. lactis* and *Lactobacillus* strains [25, 45, 46].

#### 3.1.3. *α*‐Glucosidase Inhibitory Activity

The in vitro *α*‐glucosidase inhibitory activity of the *L. lactis* isolates ranged from 25.94% to 33.96%, with no significant differences among the isolates (Table 2). This result surpasses the activity observed for *Lactobacillus sakei* MBEL1397 (3.91%), as reported by Kwun et al. [47], and is comparable to that of *Lactobacillus plantarum* 1.0665 (34.79%), reported by Jiang et al. [48], and other *L. lactis* strains (< 40%) described by Ayyash et al. [49]. The ability to inhibit *α*‐glucosidase reduces glucose absorption, thereby lowering blood glucose levels, making it a promising mechanism for preventing Type 2 diabetes [50]. The *α*‐glucosidase inhibitory activity demonstrated by the *L. lactis* isolates in this study further underscores their potential as candidates for antidiabetic applications, while acknowledging that further in vivo studies are needed to confirm their effects on blood glucose levels.

**Table 2:** *α*‐Glucosidase inhibitor activity of *L. lactis* isolates from Sui Wu′u.

Isolates	*α*‐Glucosidase inhibitor activity (%)
*L. lactis* SwR14	33.96 ± 4.83^a^
*L. lactis* SwR16	25.94 ± 8.63^a^
*L. lactis* SwR23	30.94 ± 12.14^a^
*L. lactis* SwR24	27.65 ± 4.95^a^

*Note:* Values are presented as mean and standard deviation. Different lowercase superscripts in the same column indicate significant differences (*p* < 0.05).

The *α*‐glucosidase inhibitory activity of LAB has frequently been associated with extracellular metabolites produced during bacterial growth. Previous studies have shown that cell‐free culture supernatants generally exhibit stronger *α*‐glucosidase inhibitory activity than intracellular extracts, suggesting that inhibitory compounds are predominantly released into the culture medium [51]. One proposed contributor is EPSs, as purified EPS isolated from several LAB species have demonstrated *α*‐glucosidase inhibitory activity [52]. Nevertheless, the specific extracellular compounds responsible for *α*‐glucosidase inhibition in LAB culture supernatants remain unclear and may vary among strains [51, 53]. Since the present study evaluated cell‐free culture supernatants, the observed inhibitory activity is likely attributable to extracellular metabolites produced during bacterial growth rather than intracellular components.

#### 3.1.4. Antioxidant Activity

The antioxidant activities of the *L. lactis* isolates were comprehensively evaluated using DPPH and ABTS radical scavenging assays, complemented by FRAP. Both percentage inhibition and IC_50_ values were determined to ensure a robust assessment of antioxidant potency and to overcome the limitations associated with single‐assay evaluation (Figure 2). Whereas inhibition percentage reflects scavenging effectiveness at a fixed concentration, IC_50_ values provide a quantitative measure of antioxidant efficiency, enabling more reliable comparison among isolates and with previous studies.

**Figure 2:**
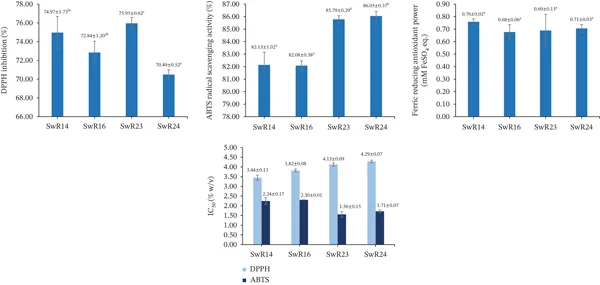
Antioxidant activity of *Lactococcus lactis* isolates assessed by DPPH and ABTS radical scavenging activity (% inhibition and IC_50_, % v/v), and ferric reducing antioxidant power (FRAP, mM FeSO_4_ equivalents). Values are expressed as mean ± standard deviation (*n* = 3). Different lowercase letters above bars indicate significant differences among isolates within the same assay (*p* < 0.05).

In the DPPH assay, all isolates exhibited strong radical scavenging activity, with inhibition values ranging from 70.49% to 75.95%. Among the isolates, SwR23 showed the highest DPPH inhibition (75.95*%* ± 0.62*%*), followed closely by SwR14 (74.97*%* ± 1.73*%*). Consistently, IC_50_ values for DPPH scavenging ranged from 3.44% to 4.29% (v/v), indicating high antioxidant potency at relatively low concentrations. SwR14 demonstrated the lowest IC_50_ value (3.44*%* ± 0.13*%* v/v), suggesting the most efficient DPPH radical scavenging activity, whereas SwR24 showed the highest IC_50_ (4.29*%* ± 0.07*%* v/v).

The ABTS assay revealed uniformly high scavenging activity across all isolates, with inhibition percentages between 82.08% and 86.05%. The highest ABTS inhibition was observed in SwR24 and SwR23, which was further supported by their low IC_50_ values. Overall, ABTS IC_50_ values ranged from 1.56% to 2.30% (v/v), with SwR23 exhibiting the strongest ABTS scavenging efficiency (1.56*%* ± 0.15*%* v/v). The consistently lower IC_50_ values obtained in the ABTS assay compared with DPPH suggest a greater capacity of the isolates to scavenge both hydrophilic and lipophilic radicals.

In contrast, the FRAP assay showed ferric reducing capacities ranging from 0.68 to 0.76 mM FeSO_4_ equivalents, with SwR14 exhibiting the highest mean reducing power. However, no statistically significant differences were observed among the isolates (*p* > 0.05), indicating comparable electron‐donating abilities.

The strong DPPH and ABTS scavenging activities, together with the absence of significant differences in FRAP values among the isolates, suggest that radical scavenging capacity plays a more prominent role than ferric reducing power in distinguishing the antioxidant profiles of the isolates. In particular, SwR14 exhibited the strongest DPPH scavenging efficiency based on IC_50_, whereas SwR23 demonstrated superior activity in the ABTS assay. This assay‐dependent variation indicates that different antioxidant mechanisms may be involved, potentially reflecting differences in the composition of bioactive metabolites produced by each isolate.

Overall, these findings confirm that the antioxidant activity of *L. lactis* is strain‐dependent and cannot be adequately characterized using a single assay. Previous studies have likewise reported considerable variability in the antioxidant activity of *L. lactis* depending on strain origin and source, including isolates from raw milk and fermented or traditional food products [54–56]. Such observations are consistent with the present results, which demonstrate assay‐dependent and isolate‐specific antioxidant activity.

### 3.2. Proteolytic Characterization of *L. lactis* Isolates

No clear zones were observed around the colonies of any tested *L. lactis* isolates on skim milk agar, indicating the absence of detectable extracellular caseinolytic activity (Figure 3). Free amino groups were subsequently quantified in fermented skim milk using the OPA assay (Table 3). SwR16 exhibited the lowest OPA value, followed by SwR14, both of which were significantly lower than the nonfermented skim milk control (*p* < 0.05). SwR23 showed an OPA value comparable to the control, whereas SwR24 exhibited a significantly higher value than all other groups (*p* < 0.05). Overall, the OPA values obtained in this study were considerably lower than those previously reported for strongly proteolytic *L. lactis* strains [12]. Together with the absence of clear zones on skim milk agar, these findings indicate that the isolates exhibit limited extracellular proteolytic activity under the conditions evaluated.

**Figure 3:**
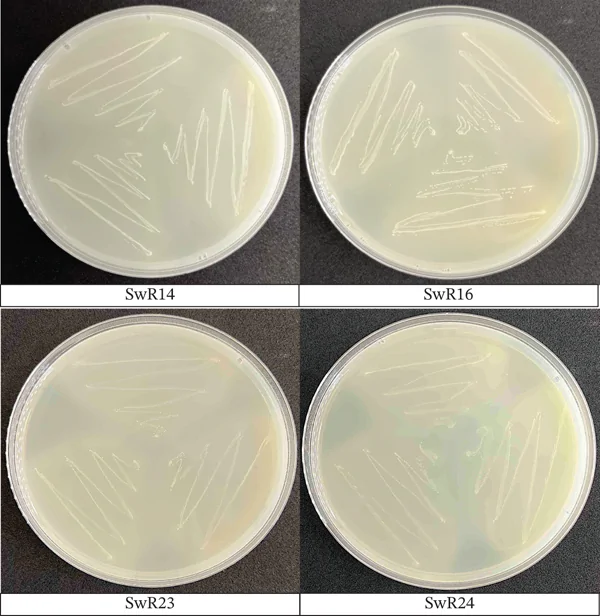
Phenotypic assessment of proteolytic activity of *Lactococcus lactis* isolates on skim milk agar. No clear zones were observed around colonies after incubation, indicating the absence of detectable extracellular caseinolytic activity. Representative plates for isolates SwR14, SwR16, SwR23, and SwR24 are shown.

**Table 3:** Free amino group content in skim milk fermented by *Lactococcus lactis* isolates as determined by the OPA assay. Values represent mean ± SD (*n* = 3). Different letters indicate significant differences (*p* < 0.05).

Sample	OPA value (OD_340 nm_)
Control (nonfermented milk)	0.068 ± 0.005^c^
SwR14	0.030 ± 0.006^ab^
SwR16	0.017 ± 0.007^a^
SwR23	0.053 ± 0.018^bc^
SwR24	0.093 ± 0.000^d^

Genotypic analysis further supported these observations, as no amplification product corresponding to the expected size (~2700 bp) of the *prtP* gene was detected in any isolate (Figure 4). The *prtP* gene encodes the CEP, a key enzyme responsible for the initial hydrolysis of milk caseins in *L. lactis* [32]. Taken together, the absence of clear zones on skim milk agar, low OPA absorbance values, and lack of detectable *prtP* amplicons indicate that the isolates examined do not rely on the classical CEP‐mediated extracellular proteolytic system for casein degradation under the conditions tested.

**Figure 4:**
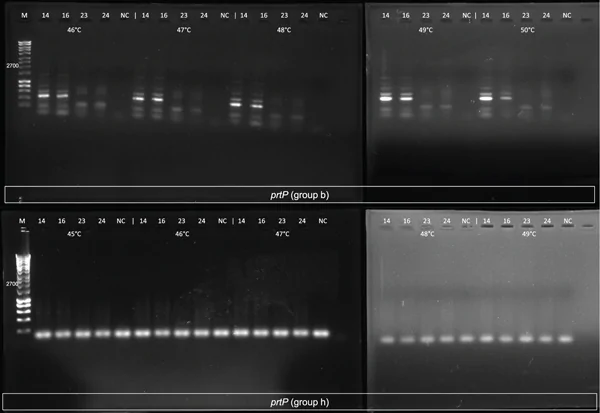
PCR amplification of the *prtP* gene (Groups b and h) in *Lactococcus lactis* isolates. Agarose gel images from four separate original gels are presented as two images for *prtP* Group b and two images for *prtP* Group h. The boundaries between the original gel images are indicated by vertical lines. M, DNA ladder; 14, SwR14; 16, SwR16; 23, SwR23; 24, SwR24; NC, negative control. Each set of lanes corresponds to PCR amplification performed at annealing temperatures of 46°C, 47°C, 48°C, 49°C, and 50°C for *prtP* Group b and 45°C, 46°C, 47°C, 48°C, and 49°C for *prtP* Group h. The original gel images were subjected only to cropping and labeling; no brightness, contrast, gamma, or other intensity adjustments were applied.

However, the proteolytic system of *L. lactis* is highly complex and strain‐dependent, comprising CEPs, peptide transport systems, and numerous intracellular peptidases that collectively determine proteolytic capacity and peptide metabolism [5, 57]. Picon et al. [12] demonstrated that efficient growth in milk may be supported by alternative peptide acquisition strategies, including oligopeptide transporters and intracellular peptidolytic activities, even in strains exhibiting limited extracellular proteolysis. Accordingly, although the isolates in this study can be classified as weakly proteolytic based on the assays employed, the contribution of alternative peptide uptake or intracellular degradation mechanisms cannot be excluded and warrants further investigation [5, 57].

From an application perspective, the absence of strong extracellular proteolytic activity does not diminish the functional relevance of these isolates. Instead, it defines a distinct physiological trait that is important for their technological and probiotic characterization. Since bioactive peptide production depends on the combined activity of extracellular proteinases, peptide transport systems, and intracellular peptidases, strains with limited extracellular proteolysis may still possess functional peptide metabolism that merits further investigation [5, 57].

### 3.3. Application of the Selected Strain in Milk Fermentation

Although none of the tested isolates showed statistically superior performance across all evaluated parameters, SwR14 was selected based on an overall assessment of all measured functional characteristics. In particular, SwR14 uniquely combined folate autotrophy under folate‐depleted conditions with the highest extracellular folate production and consistently strong antioxidant activity, while also exhibiting cholesterol‐lowering and *α*‐glucosidase inhibitory activities comparable to those of the other isolates. Therefore, SwR14 was considered the most suitable representative strain for subsequent milk fermentation and peptide characterization. Following its selection, the technological performance of *L. lactis* SwR14 was evaluated during milk fermentation. After 24 h of fermentation, SwR14 exhibited robust growth, reaching a viable count of 8.60 ± 0.22 log CFU/mL. This growth was accompanied by a decrease in pH from 6.38 to 5.41 ± 0.03 and an increase in titratable acidity to 0.68 ± 0.29, indicating active lactic acid fermentation. The soluble protein content of the fermented milk after 24 h was 0.93*%* ± 0.00*%*, as determined by the Bradford assay. The soluble protein fraction was subsequently subjected to ultrafiltration‐based peptide fractionation, followed by LC‐MS/MS analysis and evaluation of the predicted biological activities of the resulting peptide fractions.

### 3.4. Identification of Peptide Sequences in Milk Fermented With *L. lactis* SwR14

The supernatant from milk fermented with *L. lactis* SwR14 was sequentially fractionated using 10 and 3 kDa ultrafiltration membranes, yielding three fractions corresponding to nominal molecular weight ranges of > 10, 3–10, and < 3 kDa. All fractions were subsequently characterized by LC‐MS/MS. A total of 49 peptide sequences were identified across the three molecular weight fractions and subsequently profiled for their predicted biological activities using the BIOPEP‐UWM database. Among these, 47 distinct biological activities were predicted, with ACE inhibitory and DPP‐IV inhibitory properties identified in peptide sequences from all three molecular weight fractions (Figure 5). Furthermore, 33 peptide sequences were predicted to possess *α*‐glucosidase inhibitory activity, whereas 31 peptide sequences were predicted to exhibit antioxidant activity, supporting the in vitro findings of this study. Further analysis was conducted to explore their potential as novel bioactive peptides.

**Figure 5:**
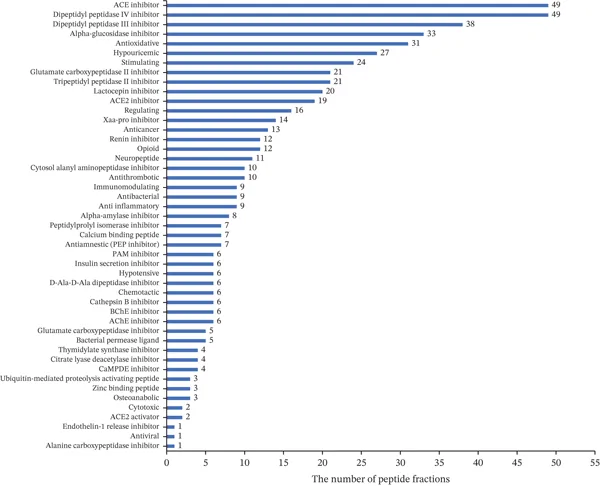
Distribution of peptide sequences exhibiting different biological activities.

Interestingly, no peptide sequences were annotated in the BIOPEP‐UWM database as cholesterol‐lowering peptides, specifically as HMG‐CoA reductase inhibitors or bile acid–binding peptides, two well‐established mechanisms underlying the hypocholesterolemic effects of food‐derived peptides [58]. However, this finding should be interpreted with caution, as the absence of annotation in BIOPEP does not necessarily indicate the absence of cholesterol‐lowering activity. For example, Soares et al. [59] identified novel HMG‐CoA reductase inhibitory peptides through experimental validation, although these peptides were not annotated as hypocholesterolemic in the BIOPEP database at the time of analysis. In addition, hypocholesterolemic peptides may exert their effects through alternative mechanisms, including modulation of intestinal cholesterol absorption via the Niemann–Pick C1‐like 1 (NPC1L1) transporter [58]. Therefore, whereas the cholesterol‐lowering activity observed in the present CFS assay is likely attributable primarily to nonpeptide metabolites or cell‐associated mechanisms, the possible contribution of currently unannotated or less‐characterized hypocholesterolemic peptides cannot be entirely excluded.

The *α*‐glucosidase inhibitory activity of peptides has been linked to their molecular weight and the presence of hydrophobic amino acids [60–63]. Ibrahim et al. [64] reported that 43 peptides with *α*‐glucosidase inhibitory activity had molecular weights less than 2000 Da (228.30–1970.23 Da). Similarly, Zhao et al. [61] reported that four peptides within 979.56–1351.78 Da exhibited stronger *α*‐glucosidase inhibition than did larger peptides. In addition, Yang et al. [63] identified 23 inhibitory peptides ranging from 475.24 to 1197.66 Da by screening those below 1300 Da. A review by Nong and Hsu [65] also highlighted that small peptides (< 5 kDa), particularly those < 3 kDa, exhibit stronger *α*‐glucosidase inhibitory activity than larger peptides do. These findings suggest that peptides smaller than 3 kDa (especially those < 2000 Da) have the potential to exhibit the highest *α*‐glucosidase inhibitory activity. This is likely due to their ability to access and bind effectively to the enzyme′s active site [63], as well as their resistance to hydrolysis during gastrointestinal digestion, which helps maintain their activity [61].

Hydrophobic amino acids also play crucial roles in *α*‐glucosidase inhibition. Mudgil et al. [66] reported that methionine, proline (Pro), phenylalanine, and leucine (Leu) contribute to enzyme binding by stabilizing interactions through hydrogen and hydrophobic bonds. Wang et al. [67] also identified several hypoglycemic peptides containing these residues, emphasizing the importance of Leu and Pro in inhibiting *α*‐glucosidase, either individually or in combination. Similarly, Ibrahim et al. [64] reported that Pro within the peptide chain and methionine at the C‐terminus enhanced inhibitory activity, reinforcing the role of hydrophobicity in enzyme inhibition. Rodhi et al. [68] further demonstrated that *α*‐glucosidase inhibitory peptides are predominantly hydrophobic, exhibiting a strong preference for amino acids with alkyl or aryl side chains, such as lysine (Lys), glutamine, Pro, and arginine (Arg), which are essential for inhibition. Zhang et al. [69] reported that *α*‐glucosidase inhibitory peptides exhibit enhanced activity when enriched with hydrophobic amino acids, particularly Leu and Pro, whereas basic residues such as Lys or Arg, especially at the N‐terminus, also contribute significantly to inhibition, primarily through hydrogen bonding interactions with the enzyme. Taken together, these findings indicate that amino acid composition plays a key role in *α*‐glucosidase inhibition, with inhibitory peptides generally exhibiting a high degree of hydrophobicity [63].

In this study, 33 peptides with predicted *α*‐glucosidase inhibitory activity were identified, with sequence lengths ranging from 8 to 24 amino acid residues. These peptide sequences were derived from *β*‐casein (13 sequences), *κ*‐casein (4 sequences), *β*‐lactoglobulin (15 sequences), and glycosylation‐related proteins (1 sequence) (Table 4). Their molecular weights ranged from 930.54 to 2521.40 Da, with all peptide sequences falling below the commonly used 3 kDa threshold for potential *α*‐glucosidase inhibitory peptides. Moreover, the repetition rate of matching hydrophobic amino acid fragments varied from 8.33% to 42.11%. Remarkably, only three peptides (GPIPNSLPQNIPPLTQTPVVVPPF, FMAIPPKK, and SLPQNIPPLTQTPVVVPPF) presented repetition rates above 30%, suggesting greater potential for *α*‐glucosidase inhibition based on BIOPEP motif occurrence. However, two of these peptide sequences are relatively large, and peptide size has been reported to influence gastrointestinal stability and access to the enzyme active site, with smaller peptides generally exhibiting stronger *α*‐glucosidase inhibitory activity [61, 65]. Therefore, experimental validation is required to confirm their *α*‐glucosidase inhibitory activity.

**Table 4:** Peptides with alpha‐glucosidase inhibitor activities.

No	Study results					BIOPEP‐UWM: Bioactive peptides		Rate of repetition^b^
	Parent protein	Sequence	Length	Molecular weight (Da)	% hydrophobic amino acid^a^	Sequence with alpha‐glucosidase inhibitor	% hydrophobic amino acid^a^	
1	*β*‐cas	GPIPNSLPQN **IPP**LTQTPVVVPPF	24	2521.40	70.83	**IPP**	100.00	12.50%
		GPIPN**SLPQNIPP**LTQTPVVVPPF				**SLPQNIPP**	62.50	33.33%
		GPIPNSLPQNI**PP**LTQTPVVVPPF				**PP**	100.00	8.33%
2	*β*‐cas	I**PP**LTQTPVVVPPF	14	1503.87	78.57	**PP**	100.00	14.29%
		**IPP**LTQTPVVVPPF				**IPP**	100.00	21.43%
3	*β*‐cas	LPQNI**PP**LTQTPVVVPPF	18	1956.11	72.22	**PP**	100.00	11.11%
		LPQN**IPP**LTQTPVVVPPF				**IPP**	100.00	16.67%
4	*β*‐cas	LV **Y** **P**FPGPIH	10	1281.63	80.00	**YP**	50.00	20.00%
5	*β*‐cas	LV **Y** **P**FPGPIHN	11	1252.66	72.73	**YP**	50.00	18.18%
6	*β*‐cas	LV **Y** **P**FPGPIHNS	12	1339.69	66.67	**YP**	50.00	16.67%
7	*β*‐cas	LV **Y** **P**FPGPIHNSLPQ	15	1677.89	66.67	**YP**	50.00	13.33%
8	*β*‐cas	MHQPHQPL**PP** T	11	1281.63	54.55	**PP**	100.00	18.18%
9	*β*‐cas	PQNI**PP**LTQTPVVVPPF	17	1843.02	70.59	**PP**	100.00	11.76%
		PQN**IPP**LTQTPVVVPPF				**IPP**	100.00	17.65%
10	*β*‐cas	**SLPQN** **IPP**LTQTPVVVPPF	19	2043.14	68.42	**SLPQNIPP**	62.50	42.11%
		SLPQNI**PP**LTQTPVVVPPF				**PP**	100.00	10.53%
		SLPQN**IPP**LTQTPVVVPPF				**IPP**	100.00	15.79%
11	*β*‐cas	V **Y** **P**FPGPIH	9	1025.53	77.78	**YP**	50.00	22.22%
12	*β*‐cas	V **Y** **P**FPGPIHN	10	1139.572	70.00	**YP**	50.00	20.00%
13	*β*‐cas	WMHQPHQPL**PP** T	12	1467.71	58.33	**PP**	100.00	16.67%
14	*κ*‐cas	FMAI**PP** KKNQD	11	1287.66	54.55	**PP**	100.00	18.18%
		FMA**IPP**KKNQD				**IPP**	100.00	27.27%
15	*κ*‐cas	FMAIPPKK	8	930.54	75.00	**PP**	100.00	25.00%
		FMAIPPKK				**IPP**	100.00	37.50%
16	*κ*‐cas	MAI**PP** KKNQD	10	1140.59	50.00	**PP**	100.00	20.00%
		MA**IPP**KKNQD				**IPP**	100.00	30.00%
17	*κ*‐cas	A**A** **D**ETWEPFASGK	13	1407.63	46.15	**AD**	50.00	15.38%
18	*β*‐lg	TPEVDD**E** **A**LEK	11	1244.57	36.36	**EA**	00.00	18.18%
		T**PE**VDDEALEK				**PE**	50.00	18.18%
19	*β*‐lg	TPEVDD**E** **A**LEKFD	13	1506.67	38.46	**EA**	50.00	15.38%
		T**PE**VDDEALEKFD				**PE**	50.00	15.38%
20	*β*‐lg	TPEVDD**E** **A**LEKFDK	14	1634.76	35.71	**EA**	50.00	14.29%
		T**PE**VDDEALEKFDK				**PE**	50.00	14.29%
21	*β*‐lg	TPEVDD**E** **A**LEKFDKA	15	1705.80	40.00	**EA**	50.00	13.33%
		T**PE**VDDEALEKFDKA				**PE**	50.00	13.33%
22	*β*‐lg	SDISLLDAQSAP**L** **R**	14	1484.78	50.00	**LR**	50.00	14.29%
23	*β*‐lg	AASDISLLDAQSAP**L** **R**	16	1626.85	56.25	**LR**	50.00	12.50%
24	*β*‐lg	AMAASDISLLDAQSAP**L** **R**	18	1828.93	61.11	**LR**	50.00	11.11%
25	*β*‐lg	LAMAASDISLLDAQSAP**L** **R**	19	1942.02	63.16	**LR**	50.00	10.53%
26	*β*‐lg	SLAMAASDISLLDAQSAP**L** **R**	20	2029.05	60.00	**LR**	50.00	10.00%
27	*β*‐lg	**V** **E**ELKPTPEGDLEILLQK	18	2050.12	50.00	**VE**	50.00	11.11%
		VEELKPT**PE**GDLEILLQK				**PE**	50.00	11.11%
28	*β*‐lg	MAASDISLLDAQSAP**L** **R**	17	1757.90	58.82	**LR**	50.00	11.76%
29	*β*‐lg	VY **V** **E**ELKPTPEGDLE	15	1716.84	46.67	**VE**	50.00	13.33%
		VYVEELKPT**PE**GDLE				**PE**	50.00	13.33%
30	*β*‐lg	LDAQSAP**L** **R**	9	969.52	55.56	**LR**	50.00	22.22%
31	*β*‐lg	VY **V** **E**ELKPTPEGDLEILLQK	20	2312.25	50.00	**VE**	50.00	10.00%
		VYVEELKPT**PE**GDLEILLQK				**PE**	50.00	10.00%
32	*β*‐lg	LKPT **PE** GDLEILLQK	15	1692.96	53.33	**PE**	50.00	13.33%
33	Glyc	ILNK **P** **E**DETH	10	1194.59	30.00	**PE**	50.00	20.00%

*Note:* Underlined amino acid sequences are considered hydrophobic. Boldface indicates peptide fragments matching *α*‐glucosidase inhibitor sequences listed in BIOPEP‐UWM. A = alanine (Ala), C = cysteine (Cys), D = aspartic acid (Asp), E = glutamic acid (Glu), F = phenylalanine (Phe), G = glycine (Gly), H = histidine (His), I = isoleucine (Ile), K = lysine (Lys), L = leucine (Leu), M = methionine (Met), N = asparagine (Asn), P = proline (Pro), Q = glutamine (Gln), R = arginine (Arg), S = serine (Ser), T = threonine (Thr), V = valine (Val), W = tryptophan (Trp), Y = tyrosine (Tyr).

Abbreviations: *β*‐lg, *β*‐lactoglobulin; Cas, casein; Glyc, glycosylation.

^a^ *%*hydrophobic amino acid = number of hydrophobic amino acid/total number of amino acid × 100.

^b^Rate of repetition = (total length of matching fragments/total length of sequence) × 100*%*.

The antioxidant activity of peptides is also closely associated with their molecular weight and the proportion of hydrophobic amino acids [70, 71]. A review by Tadesse and Emire [67] underscored that peptides with a molecular weight less than 3 kDa, particularly those containing hydrophobic amino acids such as Pro, Val, tryptophan, and phenylalanine, have been reported to demonstrate potent antioxidant properties. Xie et al. [70] reported that two high‐hydrophobicity peptide sequences, Asn‐Thr‐Val‐Pro (NTVP) and Ile‐Val (IV), were among the most potent antioxidants, exhibiting increased bioavailability, residual antioxidant activity, and gastrointestinal stability. Moreover, Zou et al. [71] reported that peptide sequences with amino acid compositions rich in hydrophobic amino acids, including glycine (Gly), alanine (Ala), Pro, Val, Leu, and isoleucine (Ile), as well as basic amino acids, such as Lys, Arg, and histidine (His), may exhibit potential antioxidant activity by inhibiting lipid peroxidation through proton donation to hydrophobic peroxyl radicals and metal ion chelation. In addition, Lys acts as a hydrogen donor, whereas His has strong radical scavenging activity, both of which may contribute to antioxidant activity.

This study identified 31 peptide sequences with predicted antioxidant activity on the basis of the BIOPEP‐UWM database (Table 5). The peptide sequences ranged from 7 to 24 amino acid residues and were derived from *α*S2‐casein (2 sequences), *β*‐casein (14 sequences), *κ*‐casein (4 sequences), *β*‐lactoglobulin (8 sequences), *α*‐lactalbumin (1 sequence), and glycosylation‐related proteins (2 sequences). Their molecular weights ranged from 836.47 to 2521.40 Da, which aligns with the recognized criterion for antioxidant peptides (< 3 kDa). The repetition rate of matching fragments ranged from 8.33% to 100%. Thirteen peptides had repetition rates above 30%, among which VGINYWLAHK showed the highest rate (100%). While this peptide exhibited the highest repetition rate, indicating the greatest sequence similarity to known antioxidant motifs in BIOPEP‐UWM, its actual efficacy remains uncertain because molecular size, gastrointestinal stability, and intestinal absorption are important determinants of the in vivo bioactivity of food‐derived peptides [58, 70, 72]. The other 12 peptides (GPIPNSLPQNIPPLTQTPVVVPPF, LVLDTDYKK, MKPWIQPK, IDALNENK, SLPQNIPPLTQTPVVVPPF, ALPMHIR, LVYPFPGPIHNSLPQ, LVYPFPGPIHNS, LVYPFPGPIHN, LVYPFPGPIH, VYPFPGPIHN, and VYPFPGPIH) also demonstrated moderate to high repetition rates (33.33%–77.78%), supporting their selection as candidates for further investigation. However, despite their high repetition rates, the relatively large molecular size of several peptide sequences may reduce gastrointestinal absorption and in vivo bioavailability [58, 70]. Therefore, experimental validation is required to confirm their antioxidant activity.

**Table 5:** Peptides with antioxidant activities.

No	Study results					BIOPEP‐UWM: Bioactive peptides		Rate of repetition^b^
	Parent protein	Sequence peptide	Length	Molecular weight (Da)	% hydrophobic amino acid^a^	Sequence with antioxidant activities	% hydrophobic amino acid^a^	
1	*α*S2‐cas	**L** **T**EEEKNR	8	1017.51	12.50	**LT**	50.00	25.00%
2	*α*S2‐cas	MK **PW**IQPK	8	1026.57	62.50	**PW**	100.00	25.00%
		MK**PWI**QPK				**PWI**	100.00	37.50%
		M**KP**WIQPK				**KP**	50.00	25.00%
		MKPWIQ**PK**				**PK**	50.00	25.00%
3	*β*‐cas	EQQQTED**EL** QDK	12	1489.652	8.33	**EL**	50.00	16.67%
4	*β*‐cas	GPIPNSLPQNIPP**L** **T**QTPVVVPPF	24	2521.40	70.83	**LT**	50.00	8.33%
		GPIPN**SLPQN** **IPP**LTQTPVVVPPF				**SLPQNIPP**	62.50	33.33%
5	*β*‐cas	IPP**L** **T**QTPVVVPPF	14	1503.87	78.57	**LT**	50.00	14.29%
6	*β*‐cas	LPQNIPP**L** **T**QTPVVVPPF	18	1956.11	72.22	**LT**	50.00	11.11%
7	*β*‐cas	LV **YPFPGPI** H	10	1281.63	80.00	**YPFPGPI**	85.71	70.00%
		L**VY**PFPGPIH				**VY**	50.00	20.00%
		LV**YP**FPGPIH				**YP**	50.00	20.00%
8	*β*‐cas	LV **YPFPGPI** HN	11	1252.662	72.73	**YPFPGPI**	85.71	63.64%
		L**VY**PFPGPIHN				**VY**	50.00	18.18%
		LV**YP**FPGPIHN				**YP**	50.00	18.18%
9	*β*‐cas	LV **YPFPGPI** HNS	12	1339.69	66.67	**YPFPGPI**	85.71	58.33%
		L**VY**PFPGPIHNS				**VY**	50.00	16.67%
		LV**YP**FPGPIHNS				**YP**	50.00	16.67%
10	*β*‐cas	LV **YPFPGPI** HNSLPQ	15	1677.89	66.67	**YPFPGPI**	85.71	46.67%
		L**VY**PFPGPIHNSLPQ				**VY**	50.00	13.33%
		LV**YP**FPGPIHNSLPQ				**YP**	50.00	13.33%
11	*β*‐cas	MHQ **PHQ** PLPPT	11	1281.63	54.55	**PHQ**	33.33	27.27%
		MHQ**PH**QPLPPT				**PH**	50.00	18.18%
12	*β*‐cas	WMHQ **PHQ** PLPPT	12	1467.71	58.33	**PHQ**	33.33	25.00%
		WMHQ**PH**QPLPPT				**PH**	50.00	16.67%
13	*β*‐cas	PQNIPP**L** **T**QTPVVVPPF	17	1843.02	70.59	**LT**	50.00	11.76%
		PQNI**PP**LTQTPVVV**PP**F				**PP**	100.00	11.76%
14	*β*‐cas	**SLPQN** **IPP**LTQTPVVVPPF	19	2043.14	68.42	**SLPQNIPP**	62.50	42.11%
		SLPQNIPP**LT**QTPVVVPPF				**LT**	50.00	10.53%
		SLPQNI**PP**LTQTPVVV**PP**F				**PP**	100.00	10.53%
15	*β*‐cas	V **YPFPGPI** H	9	1025.53	77.78	**YPFPGPI**	85.71	77.78%
		**VY**PFPGPIH				**VY**	50.00	22.22%
16	*β*‐cas	V **YPFPGPI** HN	10	1139.57	70.00	**YPFPGPI**	85.71	70.00%
		**VY**PFPGPIHN				**VY**	50.00	20.00%
17	*κ*‐cas	FMAIP**P** **K**KNQD	11	1287.66	54.55	**PK**	50.00	18.18%
18	*κ*‐cas	FMAIP**P** **K**K	8	930.54	75.00	**PK**	50.00	25.00%
19	*κ*‐cas	MAIP**P** **K**KNQD	10	1140.59	50.00	**PK**	50.00	20.00%
20	*κ*‐cas	AADE**TW** EPFASGK	13	1407.63	53.85	**TW**	50.00	15.38%
21	*β*‐lg	VE**EL** KPTPEGDLEILLQK	18	2050.12	50.00	**EL**	50.00	11.11%
		VEE**LK**PTPEGDLEILLQK				**LK**	50.00	11.11%
		VEE**LKP**TPEGDLEILLQK				**LKP**	66.67	16.67%
		VEEL**KP**TPEGDLEILLQK				**KP**	50.00	11.11%
22	*β*‐lg	VYVE**EL** KPTPEGDLE	15	1716.84	46.67	**EL**	50.00	13.33%
		**VY**VEELKPTPEGDLE				**VY**	50.00	13.33%
		**VYV**EELKPTPEGDLE				**VYV**	66.67	20.00%
		V**YVE**ELKPTPEGDLE				**YVE**	33.33	20.00%
23	*β*‐lg	IDAL **NEN**K	8	915.46	37.50	**NEN**	00.00	37.50%
24	*β*‐lg	LVLD**TDY**KK	9	1093.60	33.33	**TDY**	00.00	33.33%
		LVLDT**DYK**K				**DYK**	00.00	33.33%
25	*β*‐lg	ALPMH **IR**	7	836.47	71.43	**IR**	50.00	28.57%
		A**LPM**HIR				**LPM**	100.00	42.86%
		ALP**MHI**R				**MHI**	66.67	42.86%
26	*β*‐lg	VLVLD**TDY**KK	10	1192.67	40.00	**TDY**	00.00	30.00%
		VLVLDT**DYK**K				**DYK**	00.00	30.00%
27	*β*‐lg	VYVE**EL** KPTPEGDLEILLQK	20	2312.25	50.00	**EL**	50.00	10.00%
		VYVEE**LK**PTPEGDLEILLQK				**LK**	50.00	10.00%
		VYVEE**LKP**TPEGDLEILLQK				**LKP**	66.67	15.00%
		VYVEEL**KP**TPEGDLEILLQK				**KP**	50.00	10.00%
		**VY**VEELKPTPEGDLEILLQK				**VY**	50.00	10.00%
		**VYV**EELKPTPEGDLEILLQK				**VYV**	66.67	15.00%
		V**YVE**ELKPTPEGDLEILLQK				**YVE**	33.33	15.00%
28	*β*‐lg	**LK** PTPEGDLEILLQK	15	1692.96	53.33	**LK**	50.00	10.00%
		**LKP**TPEGDLEILLQK				**LKP**	66.67	15.00%
		L**KP**TPEGDLEILLQK				**KP**	50.00	10.00%
29	ά‐lb	**VGINYWLAHK**	10	1199.64	60.00	**VGINYWLAHK**	60.00	100.00%
		VGINYWL**AH**K				AH	50.00	20.00%
		VGINYWL**AHK**				AHK	33.33	30.00%
30	Glyc	ILN**KP** EDETH	10	1194.59	30.00	**KP**	50.00	20.00%
31	Glyc	TTEHTPSDASTTEGKLM **EL**	19	2062.93	31.58	**EL**	50.00	10.53%

*Note:* Underlined amino acid sequences are considered hydrophobic. Boldface in the “sequence peptide” column indicates peptide fragments that match the antioxidant sequences listed in the “sequence with antioxidant activities” column in BIOPEP‐UWM. A = alanine (Ala), C = cysteine (Cys), D = aspartic acid (Asp), E = glutamic acid (Glu), F = phenylalanine (Phe), G = glycine (Gly), H = histidine (His), I = isoleucine (Ile), K = lysine (Lys), L = leucine (Leu), M = methionine (Met), N = asparagine (Asn), P = proline (Pro), Q = glutamine (Gln), R = arginine (Arg), S = serine (Ser), T = threonine (Thr), V = valine (Val), W = tryptophan (Trp), Y = tyrosine (Tyr).

Abbreviations: ά‐lb, ά‐lactalbumin; *β*‐lg, *β*‐lactoglobulin; Cas, casein; Glyc, glycosylation.

^a^ *%*hydrophobic amino acid = number of hydrophobic amino acid/total number of amino acid × 100.

^b^Rate of repetition = (total length of matching fragments/total length of sequence) × 100*%*.

## 4. Conclusions

This study demonstrates that indigenous *L. lactis* isolates from Sui Wu′u exhibit strain‐dependent variability in nutritional and functional traits. Among the tested isolates, SwR14 demonstrated folate autotrophy under folate‐depleted conditions, extracellular folate production, antioxidant activity, and moderate in vitro cholesterol‐lowering and *α*‐glucosidase inhibitory activities. Although SwR14 exhibited a weak proteolytic phenotype, milk fermentation generated multiple soluble peptide sequences with predicted antioxidant and *α*‐glucosidase inhibitory activities. However, these peptide bioactivities were inferred solely through in silico analysis and were not experimentally validated. In addition, fermentation was evaluated under a single set of conditions, which may not reflect the full metabolic potential of the strain. Therefore, the present findings should be interpreted within these limitations. Future studies should experimentally validate the identified peptide sequences using enzyme‐ and cell‐based assays, optimize fermentation conditions to enhance peptide production, and perform in vivo studies to evaluate their physiological relevance.

## Author Contributions

Conceptualization: Y.T.R., H.J.D.L., and M.U.E.S.; methodology: Y.T.R.; formal analysis and investigation: Y.T.R., F.A.M., and D.Y.; writing—original draft preparation: Y.T.R., F.A.M., and D.Y.; writing—review and editing: Y.T.R., F.A.M., D.Y., H.J.D.L., and M.U.E.S.; funding acquisition: Y.T.R.; resources: Y.T.R.; supervision: Y.T.R., F.A.M., D.Y., H.J.D.L., and M.U.E.S.

## Funding

This work was supported by the Ministry of Education, Culture, Research, and Technology, Republic of Indonesia (043/E5/PG.02.00.PT/2023).

## Conflicts of Interest

The authors declare no conflicts of interest.

## Data Availability

All the data generated or analyzed during this study are included in this published article.
